# Reflections of Oneself: Neurocognitive Evidence for Dissociable Forms of Self-Referential Recollection

**DOI:** 10.1093/cercor/bhu063

**Published:** 2014-04-03

**Authors:** Zara M. Bergström, David A. Vogelsang, Roland G. Benoit, Jon S. Simons

**Affiliations:** 1 Department of Psychology, University of Cambridge, Downing Street, Cambridge CB2 3EB, UK; 2 Behavioural and Clinical Neuroscience Institute, University of Cambridge, Downing Street, Cambridge CB2 3EB, UK; 3 School of Psychology, Keynes College, University of Kent, Canterbury CT2 7NP, UK; 4 Department of Psychology, Harvard University, Cambridge, MA 02138, USA

**Keywords:** episodic retrieval, fMRI, medial PFC, self, social cognition

## Abstract

Research links the medial prefrontal cortex (mPFC) with a number of social cognitive processes that involve reflecting on oneself and other people. Here, we investigated how mPFC might support the ability to recollect information about oneself and others relating to previous experiences. Participants judged whether they had previously related stimuli conceptually to themselves or someone else, or whether they or another agent had performed actions. We uncovered a functional distinction between dorsal and ventral mPFC subregions based on information retrieved from episodic long-term memory. The dorsal mPFC was generally activated when participants attempted to retrieve social information about themselves and others, regardless of whether this information concerned the conceptual or agentic self or other. In contrast, a role was discerned for ventral mPFC during conceptual but not agentic self-referential recollection, indicating specific involvement in retrieving memories related to self-concept rather than bodily self. A subsequent recognition test for new items that had been presented during the recollection task found that conceptual and agentic recollection attempts resulted in differential incidental encoding of new information. Thus, we reveal converging fMRI and behavioral evidence for distinct neurocognitive forms of self-referential recollection, highlighting that conceptual and bodily aspects of self-reflection can be dissociated.

## Introduction

Much cognitive neuroscience research indicates a strong link between social cognition and the medial prefrontal cortex (mPFC) across various tasks and cognitive domains (Amodio and Frith 2006; Mitchell 2009). Within the mPFC, a dorsal–ventral functional gradient has often been observed (Northoff and Bermpohl 2004; Moran et al. 2011). Whereas the dorsal mPFC is linked with processing social information about people in general (e.g., Hassabis et al. 2013), the ventral mPFC is more engaged when processing information in relation to oneself (Mitchell et al. 2006; D'Argembeau et al. 2007; Denny et al. 2012; Wagner et al. 2012). Furthermore, activation in the ventral mPFC is most often elicited in tasks that involve processing conceptual as opposed to bodily aspects of the self (e.g., reflections on one's personality traits rather than judgments of agency), whereas the latter tends to be associated with activation in sensorimotor and posterior cortical regions (Blakemore and Frith 2003; Gillihan and Farah 2005; Powell et al. 2010). Hence, the ventral mPFC may be a core component in a network of medial cortical regions that are specifically dedicated to conceptual self-referential processing (e.g., Gusnard et al. 2001; Kelley et al. 2002; Powell et al. 2010; Martinelli et al. 2013; although see Roy et al. 2012).

Episodic memories—recollections of personally experienced events—are intrinsically self-related, and are critical for our sense of a coherent identity that extends across time (Schacter et al. 2007). However, episodic memory may involve different degrees or types of self-referential processing, depending both on how information is initially encoded as well as on the type of information oriented towards during subsequent retrieval (e.g., Summerfield et al. 2009). During episodic encoding, relating information to one's concept of self (e.g., “Does the word *intelligent* describe you?”) improves later memory and is associated with greater ventral mPFC activity compared with relating the same information to another person (e.g., “Does the word *intelligent* describe President George Bush?”; Kelley et al. 2002). Furthermore, the level of activation in ventral mPFC predicts the magnitude of the later memory advantage for self-encoded items (Macrae et al. 2004; see also Leshikar and Duarte 2011). Another type of self-related processing—interacting with the world as an agent as opposed to watching another person perform an action—also enhances memory encoding (e.g., Cohen 1983; Engelkamp and Zimmer 1989). However, this enactment-based memory effect has been related to the involvement of motor-planning regions during encoding of self-performed actions rather than the mPFC (Powell et al. 2010), consistent with a distinction between bodily and conceptual aspects of self (see also Lind 2010).

During episodic retrieval, remembering conceptual information that was encoded in relation to oneself as opposed to another person activates the mPFC (Fossati et al. 2004; Benoit et al. 2010). In contrast, remembering self-performed actions compared with verbal descriptions of actions activates sensorimotor regions (e.g., Nyberg et al. 2002). Such similarity between cortical activation at encoding and retrieval is predicted by the transfer-appropriate processing framework, which proposes that successful retrieval involves the reinstatement of neurocognitive processes that were active at the time of encoding (Morris et al. 1977; Rugg et al. 2008). Interestingly, asking participants to remember whether they or another person performed an action activates the mPFC compared with other types of memory judgments (Brandt et al. 2014; Vinogradov et al. 2006; Simons et al. 2008). This finding raises the possibility that orienting towards *social* agency information (“was it me or you?”) during retrieval judgments engages mPFC-mediated cognitive processes that are not automatically elicited during retrieval of self-performed actions without a social element (cf. Nyberg et al. 2002). However, to our knowledge, no previous study has tested the extent to which agentic versus conceptual self/other judgments in recollection involve the recruitment of the same neural system, a question that formed the main focus of the current study.

We also tested another, complementary prediction related to the transfer-appropriate processing framework, namely that if retrieval involves reinstating the neurocognitive processes engaged when initially encoding an experience, then each retrieval attempt is potentially also an encoding event. Hence, if participants reinstate distinct types of neurocognitive processes depending on the type of information they are trying to retrieve, this may in turn affect the incidental encoding of novel information presented in a retrieval test. Previous research has shown that new “foil” words presented during an old/new recognition test tend to be incidentally encoded, as assessed by a surprise subsequent recognition test for the foils, and that such incidental encoding is enhanced if the foils are first encountered intermixed with semantically encoded old words compared with phonologically encoded old words (Jacoby, Shimizu, Daniels, et al. 2005; Jacoby, Shimizu, Velanova 2005). Because semantic processing typically leads to enhanced memory compared with phonological processing (Craik and Tulving 1975), this finding suggests that people strategically reinstate a semantic processing mode when attempting to retrieve semantically encoded information, and a phonological processing mode when attempting to retrieve phonologically encoded information (see also Marsh et al. 2009; Danckert et al. 2011). In the current study, we examined whether orienting retrieval towards the conceptual self would lead to differential encoding of new information compared with orienting retrieval towards the agentic self, as might be expected if these types of processing modes are distinct.

These questions were addressed in an fMRI experiment involving source judgments about agentic or conceptual self-referential memory. During study, participants viewed lists of person-descriptive words that were first read out loud either by the participant or the experimenter. Next, participants judged how well the person-descriptive word applied either to themselves or the President of the USA, Barack Obama (Kelley et al. 2002). During a subsequent scanned test, participants were presented with the words again, intermixed with new “foil” words. Participants were asked on a trial-by-trial basis to remember either whether they themselves or the experimenter had spoken the word, or whether it was new (orienting retrieval towards agentic self/other information); or, whether they had related the word to themselves or Obama, or whether it was new (orienting retrieval towards conceptual self/other information). A third nonepisodic control condition was also included. Following scanning, a surprise recognition test assessed whether agentic and conceptual recollection attempts had led to different degrees of incidental encoding of foils.

We predicted that both types of episodic recollection task would activate dorsal mPFC compared with the nonepisodic control condition, since both episodic tasks involve considering social information. In contrast, since the ventral mPFC is particularly linked to conceptual self-referential processing, we expected enhanced activation during recollection of person-descriptive words that participants had related to themselves rather than Obama during study. Finally, engaging such distinct self-referential retrieval processes when asked to retrieve conceptual versus agentic information, may, in turn, result in encoding differences of new items that served as lures. In that case, participants were expected to show differences in subsequent recognition of foil items from the conceptual versus agentic retrieval task.

## Materials and Methods

### Participants

Eighteen right-handed healthy native English speakers (5 male, 13 female, mean age = 22.5 years, and range 19–29), with normal or corrected to normal vision were screened using a comprehensive medical questionnaire and gave written informed consent before entering the MRI scanner. Participants received £30 in compensation for taking part. The study was approved by the University of Cambridge Psychology Research Ethics Committee.

### Materials

Stimuli consisted of 288 person-descriptive words (e.g., “gentle, jealous, bossy”) derived from Dumas et al. (2002). These words were split into 16 lists of 18 items each that were matched for word length, likableness, familiarity, and Kucera–Francis word frequency (Wilson 1988). List assignment to conditions was fully counterbalanced across participants.

### Design and Procedure

After initial practice of one study and one test phase, participants completed 16 study-test cycles in the scanner. Each study-test cycle was ∼2.5 min, meaning that the total time in the scanner was ∼40 min, plus short breaks. Only the test phases were scanned to avoid movement artifacts in the fMRI data due to the participant speaking during the study phase.

**Figure 1.**
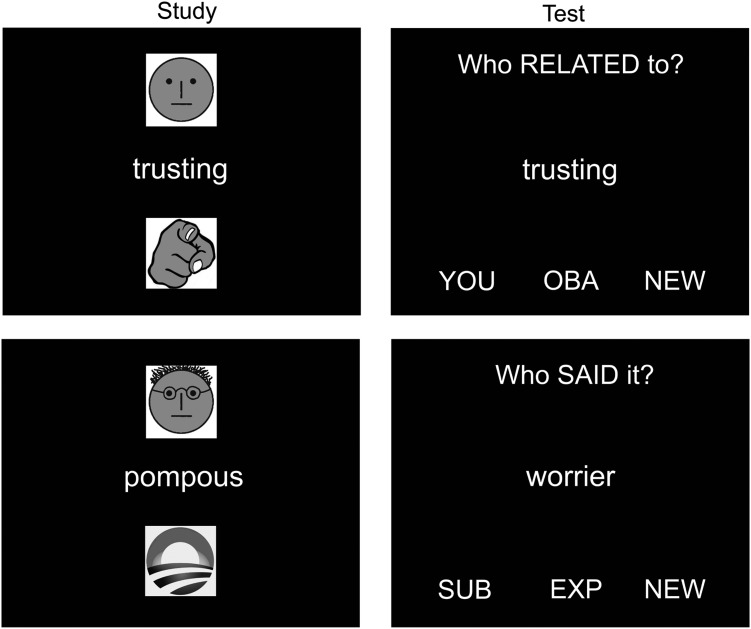
Stimuli examples in the study (left column) and test (right column) phases. In the study phase, a symbol at the top of the screen indicated whether the participant (a “plain” face) or the experimenter (a face resembling the experimenter) should speak the word out loud. A symbol at the bottom of the screen indicated whether participants should judge how well the word reflected themselves (pointing hand) or the US President Obama (the “Obama 2008” campaign logo). In the test phase, a question at the top of the screen indicated to participants whether they should remember who had spoken the word at study (Agentic recollection), remember who the word had been related to at study (Conceptual recollection), or make a nonepisodic Control judgment. Top left: a word spoken by the participant at study (Subject) that they also related to themselves (You). Top right: the same word tested with the Conceptual recollection question. Bottom left: a word spoken by the Experimenter at study that the participant related to Obama. Bottom right: a new word tested with the Agentic recollection question.

A study phase consisted of 8 trials. Each trial began with a 500 ms fixation cross followed by a person-descriptive word that appeared in the center of the screen for 500 ms, after which a cue at the top of the screen appeared for 3000 ms indicating who was to read out the word, either the participant or the experimenter (indicated by a “neutral” or an “experimenter” face symbol respectively, see Fig. 1). Participants spoke the word out loud on Subject trials and listened to the experimenter speaking the word over the intercom in Experimenter trials. Subsequently, a second cue appeared at the bottom of the screen for another 3000 ms indicating whether the participant had to judge the extent to which the person-descriptive word applied to either him/herself or to Obama (indicated by a “pointing hand” or an “Obama 2008” symbol respectively, see Fig. 1). Participants made their judgment via 4 buttons with their right hand (“sure no”, “unsure no”, “unsure yes”, and “sure yes”). These 2 study phase factors were fully crossed, and 2 trials of each possible combination were presented in a pseudorandom order (not more than 3 repetitions of the same condition) in each study phase.

The subsequent source memory test phases comprised 18 trials each, in which participants were presented with person-descriptive words in the center of the screen that had either previously been seen or were new, and were asked one of 3 questions. Six trials in each phase assessed memory for whether items had been read out loud by the participant or the experimenter during study, or whether they were new (the “Agentic condition” or “Agentic task”), with 2 items of each old type and 2 new foils); 6 assessed memory for whether items had been related to the participant or to Obama during study, or whether they were new (the “Conceptual condition” or “Conceptual task”), with 2 items of each old type and 2 new foils); and 6 trials required participants to judge the number of letters in novel personality trait words (the “Control condition” or “Control task”). The order of test questions was pseudorandom to ensure that the same question was not repeated more than 3 times in a row, and that the last item presented during the preceding study phase was not the first item presented at test.

Each trial began with a 475 ms fixation cross, after which one of the 3 questions appeared at the top of the screen (“Who Said it?” for Agentic recollection, “Who Related to?” for Conceptual recollection, or “Letters?” for Control), for a total duration of 4500 ms. One second after the question appeared, the person-descriptive word appeared in the center of the screen for 3500 ms, and participants were required to respond within this time (see Fig. 1). For each question, symbols at the bottom of the screen indicated the 3 different answers participants could choose between (Agentic recollection: “SUB”, subject said it; “EXP”, experimenter said it; “NEW”, the word is new. Conceptual recollection: “YOU”, related it to themselves; “OBA”, related it to Obama; “NEW”, the word is new. Control condition: “<8”, fewer than 8 letters; “8–10” between 8 and 10 letters; “>10”, more than 10 letters. These word length values were chosen since approximately one-third of items fell within each category). In all conditions, participants were instructed to use the duration of their button press to indicate their levels of confidence in their answer, with higher confidence indicated by a longer button press. When pressing a button, the font color of the chosen option would initially turn green, and the font color would change from green towards red with continued button press.

### Postscan Foil Recognition Test

After completing the experimental phases, participants undertook a surprise test outside of the scanner, which assessed their recognition memory for foils that had been previously presented during the test phase of the prior experiment. All previously seen foil words from the Agentic and Conceptual test conditions (32 items in each condition, 2 from each of the preceding 16 test phases) were pseudorandomly intermixed with completely new person-descriptive words (64 items), and were presented for 3500 ms in the center of the screen. The new person-descriptive words for the foil test were also selected from Dumas et al. (2002) and had similar characteristics to the words in the main experiment, but were not counterbalanced across the other conditions because our hypothesis only concerned differences in recognition performance for foils that had been previously seen in the agentic versus conceptual source memory tests. Participants were instructed to judge a word as “old” if it had been presented earlier in any phase of the experiment, and to respond “new” if the word had not been presented during any phase of the experiment. Responses were given on a 4-point confidence scale (“sure old”, “unsure old”, “unsure new”, and “sure new”).

### Postscan Similarity Test

Finally, following the foil recognition test, participants undertook a test to measure their perceived personality similarity with President Obama. In this similarity test, participants made judgments about Obama using the person-descriptive words that they had previously related to themselves in the study phase and vice versa. In this way, participants provided both a self and an Obama rating for each word.

In order to measure the extent to which participants perceived themselves as similar to President Obama, Pearson correlation coefficients were computed between the self and Obama judgments. These served as a similarity index, and were Fisher *Z* transformed. It has been suggested that self-referential processes are also applied when considering someone who is perceived as similar to the self (e.g., Mitchell et al. 2006). As a corollary, one would engage similar encoding processes when judging oneself and that similar other person, which would result in less discriminable memory traces (Benoit et al. 2010). Thus, individuals who perceived themselves as more similar to Obama should be worse at remembering whether they had related a word to themselves or Obama during study (Benoit et al. 2010).

### FMRI Data Acquisition and Analysis

Structural MPRAGE images and functional images were acquired with a 3T Siemens Allegra system (repetition time = 2250 ms, echo time = 30 ms, 36 interleaved axial slices oriented ∼10–20° from the AC–PC transverse plane, 2 mm thickness, 1 mm interslice skip, 192 mm field of view [FOV], 64 × 64 matrix). In order to allow for *T*_1_ equilibration, the first 4 volumes from each session were discarded.

Data were preprocessed and analyzed using SPM8 (Welcome Department of Imaging Neuroscience, London). All acquired images for each participant were realigned with respect to the first for motion correction and all slices were resampled in time to match the middle slice. Participants' structural scans were coregistered to their mean functional image, and the coregistered structural scan was segmented to separate out gray matter and generate normalization parameters. Next, these normalization parameters were used to normalize the realigned and slice-timing corrected functional images into 3-mm cubic voxels in Montreal Neurological Institute (MNI) stereotactic space (Cocosco et al. 1997). The normalized images were then spatially smoothed with an 8-mm full-width at half-maximum (FWHM) isotropic Gaussian kernel.

Statistical analysis of random effects was undertaken in 2 stages. In the first stage, the 16 sessions were concatenated and delta functions representing onset times of the experimental conditions of interest were convolved with a canonical hemodynamic response function after discarding onsets that fell in the last 16 s of a session since their response could not be accurately modeled. Sessions were concatenated due to the low number of trials in each functional run to ensure an adequate trial number for the estimation of each regressor (e.g., Benoit et al. 2010). A subject-level model was used to estimate the parameters for each regressor, with movement parameters in the 3 directions of motion and 3 degrees of rotation included as vectors of no interest to avoid movement confounds. However, with concatenated functional runs, it is not possible to use the standard SPM high-pass filter, which would treat the runs as one continuous time-series. We thus included the following regressors in order to control for temporal drifts and for run-specific mean activation levels: a linear-trend predictor, a 6-predictor Fourier basis for nonlinear trends (sines and cosines of up to 3 cycles per run) and a confound-mean predictor (Kriegeskorte et al. 2008).

For the episodic tasks, 6 separate regressors coded the onsets of: 1) old items in the Agentic recollection task that participants had spoken during study and that received an accurate source judgment (the “Subject” condition); 2) old items in the Agentic recollection task that the experimenter had spoken during study and that received an accurate source judgment (the “Experimenter” condition); 3) correctly identified New items in the Agentic recollection task; 4) old items in the Conceptual recollection task that participants had related to themselves during study and that received an accurate source judgment (the “You” condition); 5) old items in the Conceptual recollection task that participants had related to Obama during study and that received an accurate source judgment (the “Obama” condition); 6) correctly identified New items in the Conceptual recollection task. Regressors 7–10 consisted of new items in the Control task that received an accurate letter number judgment and that were randomly split into quarters and modeled with 4 separate regressors. This split was implemented because one analysis of interest involved investigating common activation for both old and new items during both episodic tasks compared with the Control condition, thus splitting Control trials into 4 allowed each episodic condition to be compared against an independent baseline. A final 11th regressor coded old and new items from any task for which participants gave no response or the wrong response with the purpose of removing noise variability from the first level statistical model.

In a second analysis, another first level model was created to investigate differences between old items in the different recollection tasks based on whether the participant had related a word to themselves or Obama during study, irrespective of whether the experimenter or participant had spoken the word out loud. This analysis included the same regressors as above with the exception that old items were grouped according to You versus Obama study condition in both retrieval tasks.

In the second stage of each analysis, the beta estimates from the first level were entered into a general linear model treating subjects as a random effect. The mPFC was defined as an a priori region of interest. Coordinates of the anterior rostral medial PFC region identified by Amodio and Frith (2006) as specifically sensitive to social cognition were used to create a mask for small-volume correction. The mask had a posterior boundary at the edge between the corpus callosum and the anterior cingulate cortex (approximate *Y* coordinate 31), left and right boundaries at MNI *X* coordinates −15 and 15, respectively, the lower boundary on the horizontal plane approximately at MNI *Z* coordinate 4, and an upper boundary line passing through approximate MNI points (50, 41) and (30, 15) as defined by Steele and Lawrie (2004, see Fig. 5). This mask was smoothed with a 8 mm FWHM kernel, and activations within this mPFC region were characterized using an uncorrected height threshold of *P* < 0.001 with a minimum cluster size of 10 voxels, reported as significant when the peak exceeded the small-volume corrected family-wise error threshold of *P* < 0.05. Activations outside the mPFC were reported when they were significant at *P* < 0.05 whole-brain family-wise error corrected, with a minimum cluster size of 10 voxels. The approximate Brodmann's areas of significant clusters were estimated using the Talairach and Tournoux (1988) atlas and the Talairach daemon software, after adjusting coordinates to allow for differences between the MNI and Talairach templates using a nonlinear transform (http://imaging.mrc-cbu.cam.ac.uk/imaging/MniTalairach).

## Results

### Source Memory Test Results

Table 1 shows the behavioral data from the scanned retrieval test phases. Note that old recognition rate was calculated as the proportion of items in old conditions that were attributed to either of the 2 sources irrespective of source accuracy. For source accuracy, we used the raw proportion accurate responses for each condition. In the first step, we tested for typical self-referential effects, whereby items that participants had related to themselves or spoken out loud were predicted to be associated with higher memory accuracy than items that participants had related to Obama or that had been spoken by the experimenter. Planned comparisons confirmed that self-encoded conditions were associated with significantly higher old recognition rate than other-encoded items in both memory tasks (Subject vs. Experimenter: *t*_(17)_ = 2.87, *P* = 0.011; You vs. Obama: *t*_(17)_ = 2.21, *P* = 0.042), in line with a large body of previous research. However, there were no significant differences between Subject- and Experimenter-spoken items in the Agentic recollection task for source accuracy (proportion correct responses), RT or Confidence (all *P* > 0.14). You- and Obama-related items in the Conceptual recollection task were also not significantly different for source accuracy (*t* < 1, ns), but You items were associated with significantly shorter RTs (*t*_(17)_ = 4.52, *P* = 0.0003) and higher Confidence (*t*_(17)_ = 4.12, *P* = 0.0007) than Obama items.

**Table 1:** Memory test performance

	Proportion accurate		Reaction time (ms)		Confidence time (ms)		Proportion old recognition rate	
	Mean	SEM	Mean	SEM	Mean	SEM	Mean	SEM
Agentic								
Subject	0.63	0.04	1970	74	433	35	0.94	0.01
Experimenter	0.71	0.03	2023	54	406	31	0.90	0.01
New	0.96	0.01	1346	74	683	52		
Conceptual								
You	0.77	0.03	1987	80	531	49	0.93	0.01
Obama	0.75	0.03	2163	60	455	40	0.91	0.01
New	0.97	0.01	1398	77	681	53		
Control	0.87	0.02	1622	87	601	39		

Next, we compared performance across the 2 recollection tasks. This analysis was important for interpreting any putative differences between foils on the subsequent foil recognition task, because previous research has shown that later recognition tends to be more accurate for foils that are presented intermixed with studied items that are more accurately remembered in the initial retrieval test (e.g., Jacoby, Shimizu, Daniels et al. 2005; Jacoby, Shimizu, Velanova 2005). Comparing all old items in the Agentic and Conceptual tasks against each other, irrespective of study conditions, showed that both source accuracy and confidence was significantly higher for Conceptual source memory judgments than Agentic source memory judgments (accuracy: *t*_(17)_ = 3.20, *P* = 0.005; confidence: *t*_(17)_ = 2.87, *P* = 0.011). However, the 2 memory tasks did not differ in reaction time (*t*_(17)_ = 1.63, *P* = 0.12). There were no significant differences between Agentic and Conceptual new items on any measure (accuracy: *t*_(17)_ = 1.37, *P* = 0.19; RT: *t*_(17)_ = 1.72, *P* = 0.10; confidence: *t*_(17)_ < 1, ns). Thus, performance for new foils was highly similar across tasks in the source memory test, suggesting that any subsequent differences on the foil recognition test (next section) cannot be simply explained by differences in processing effort or study time during initial encoding in the first test.

Performance in both recollection tasks was also compared against performance in the Control task. This analysis assessed whether potential behavioral differences between memory and Control tasks could explain the fMRI activations seen for both old and new items in the episodic retrieval tasks when compared with the Control condition (see fMRI Results section). Previously seen items in the recollection tasks were associated with lower accuracy (Agentic Subject: *t*_(17)_ = 5.52, *P* = 0.00004; Agentic Experimenter: *t*_(17)_ = 3.72, *P* = 0.002; Conceptual You: *t*_(17)_ = 2.93, *P* = 0.009; Conceptual Obama: *t*_(17)_ = 3.35, *P* = 0.004) and longer reaction times (Agentic Subject: *t*_(17)_ = 3.94, *P* = 0.001; Agentic Experimenter: *t*_(17)_ = 5.33, *P* = 0.00006; Conceptual You: *t*_(17)_ = 4.19, *P* = 0.001; Conceptual Obama: *t*_(17)_ = 7.38, *P* = 0.000001) than the Control condition. Old items were also associated with significantly lower confidence than Control items (Agentic Subject: *t*_(17)_ = 4.30, *P* = 0.0005; Agentic Experimenter: *t*_(17)_ = 6.28, *P* = 0.000008; Conceptual Obama: *t*_(17)_ = 4.93, *P* = 0.0001), with the exception of items that the participant had related to themselves during study, which only showed a trend for lower confidence (Conceptual You: *t*_(17)_ = 1.89, *P* = 0.08). In contrast, new items in both recollection tasks were associated with higher accuracy (Agentic New: *t*_(17)_ = 3.38, *P* = 0.004; Conceptual New: *t*_(17)_ = 4.37, *P* = 0.0004) and confidence ratings (Agentic New: *t*_(17)_ = 2.45, *P* = 0.026; Conceptual New: *t*_(17)_ = 2.33, *P* = 0.032) and shorter reaction times (Agentic New: *t*_(17)_ = 3.95, *P* = 0.001; Conceptual New: *t*_(17)_ = 2.96, *P* = 0.009) than the Control condition. This behavioral pattern thus means that any common episodic task effects on brain activity that occurred for both old and new items compared with the Control task cannot be explained by simple differences in accuracy, RT, or confidence.

Consistent with prior research, participants who thought of themselves as more alike to Obama (as shown by the similarity index) were significantly less accurate at remembering whether a word had been related to themselves or Obama (*r*_(16)_ = −0.53, *P* = 0.024). By comparison, there was no significant relationship between the similarity measure and performance on the Agentic recollection task (*r*_(16)_ = −0.15, *P* = 0.56). The difference between these correlation coefficients was at trend-level according to a Williams *t*-test for dependent correlations (*t*_(15)_ = 1.49, *P* = 0.08, one-tailed). Thus, consistent with the hypothesis that one would also engage self-referential (encoding) processes when thinking about someone similar to oneself (Mitchell et al. 2006; Benoit et al. 2010), people who perceived themselves as more similar to Obama found it more difficult to subsequently remember whether they had in fact made conceptual judgments about themselves or Obama.

### Foil Recognition Results

Despite highly similar behavioral performance for foils during their initial exposure, they were not remembered equally well on a subsequent foil recognition test. The hit rate was significantly higher for foils that had been presented with a Conceptual than Agentic retrieval question during the preceding source memory test (Conceptual: mean proportion correct = 0.66, SEM = 0.03; Agentic: mean proportion correct = 0.57, standard error of the mean (SEM) = 0.03; *t*_(17)_ = 3.18, *P* = 0.005). RT and confidence measures did not differ across the 2 types of foils (Conceptual: mean RT (ms) = 1745, SEM = 307; Agentic: mean RT (ms) = 1715, SEM = 207; Conceptual: mean proportion confident responses = 0.45, SEM = 0.04; Agentic: mean proportion confident responses = 0.44, SEM = 0.04; both ts < 1, ns). Thus, the results support the hypothesis that retrieval of conceptual versus agentic self-referential information rely on different neurocognitive processes.

### FMRI Results

We first discuss the simple retrieval effects (episodic task effects and basic old/new effects) and then turn to the critical and more complex analysis of the activation differences as a function of both study condition and retrieval task.

### General Episodic Task Effects Compared with Control

The first fMRI analysis investigated common effects of episodic retrieval instructions compared with the Control condition, irrespective of retrieval task and memory status (old vs. new) of the items. A conjunction analysis was conducted on individual contrasts between Agentic old > Control, Agentic new > Control, Conceptual old > Control and Conceptual new > Control. Because these contrasts used the same Control condition they were not statistically independent, which can bias the conjunction statistical test. Therefore, it would be inappropriate to report the *t*-values generated by this nonindependent conjunction analysis. Instead, common activation across contrasts was defined as regions where each effect was independently significant at the specified threshold, and the conjunction analysis was only used to localize the peaks in this overlap. *T*-values for those peaks were reported for each individual contrast.

**Figure 2.**
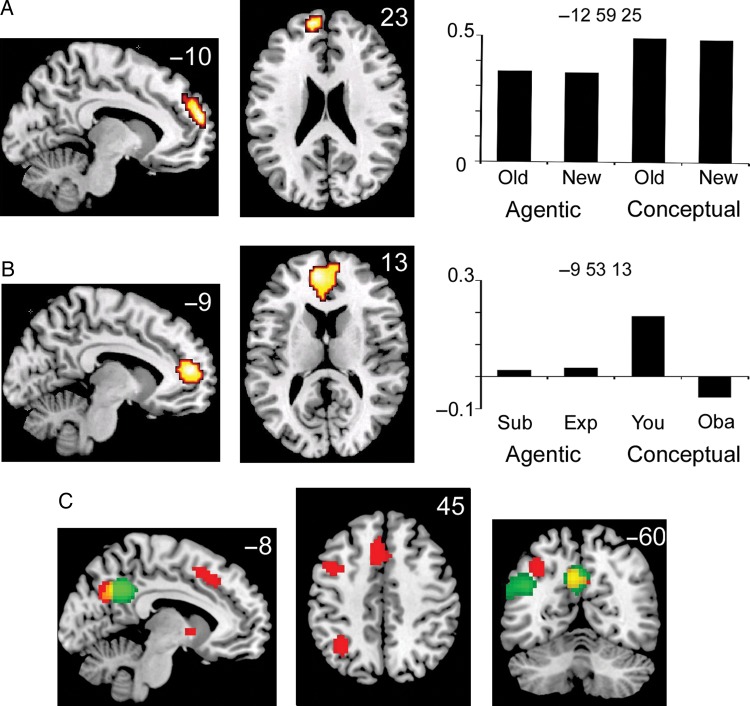
fMRI activations in the mPFC associated with self/other recollection. Effects in *A* and *B* are thresholded at *P* < 0.001 (uncorrected), with a minimum cluster size of 10 voxels, and inclusively masked to display only activations within the mPFC region associated with social cognition in Amodio and Frith (2006). Effects in (*C*) are thresholded at *P* < 0.05 family-wise error corrected for the whole brain, with a minimum cluster size of 10 voxels. The percent signal change bar graphs (*A* and *B*) plot the mean difference between each displayed condition and the nonepisodic Control task extracted from the peak voxel in each mPFC cluster. (*A*) A dorsal mPFC region with a peak at [−12, 59, 25] showed enhanced activation for both old and new items in both recollection tasks, compared with the Control condition. (*B*) a ventral mPFC region with a peak at [−9, 53, 13] showed selective activation for old items that participants had processed in relation to their conceptual self during study, and only when the retrieval task required recollection of conceptual self/other information. (*C*) In a whole-brain analysis, general old > new effects (old > new collapsed across retrieval task; red) were associated with a very different activation pattern from episodic retrieval task effects (episodic tasks > Control task; green), except in the precuneus where the 2 effects overlapped.

A region of interest (ROI) analysis focusing on the mPFC region that has previously been implicated in social cognition (using a mask based on coordinates identified by Amodio and Frith (2006) for small-volume correction) confirmed that all episodic conditions did indeed activate a relatively dorsal cluster in the left mPFC (Table 2; Fig. 2*A*). An exploratory analysis testing for regions outside the mPFC that were commonly activated for old and new items in both retrieval tasks showed further activations in the medial parietal and left lateral temporo-parietal cortex, left inferior frontal and left middle temporal regions (Table 2).

**Table 2:** fMRI activations associated with episodic retrieval mode that were common across retrieval task and old/new item memory status

Hemisphere	Region	BA	*x*	*y*	*z*	Voxels	Agentic old > Control *T*-value	Agentic new > Control *T*-value	Conceptual old > Control *T*-value	Conceptual new > Control *T*-value	Independ-ent contrasts conjunct-ion *T*-value
Medial PFC ROI											
Left	Superior frontal gyrus	10	−12	59	25	68	4.87	4.72	6.44	5.45	3.73
Whole-brain analysis											
Bilateral	Precuneus/posterior cingulate cortex	7/23/31	−6	−55	37	427	14.56	10.88	14.26	10.33	8.79
Left	Middle temporal gyrus/superior temporal gyrus	21/22	−57	−37	1	194	7.72	7.95	9.65	8.29	6.71
Left	Inferior frontal gyrus	47/45/46	−45	29	−5	146	8.77	8.07	9.49	7.58	6.49
Left	Superior temporal gyrus/angular gyrus	39	−42	−61	28	251	9.39	7.33	10.82	7.3	5.61

BA, approximate Brodmann area.

Notes: Activation within the mPFC was initially height thresholded at *P* < 0.001 uncorrected, >10 voxels and subsequently small-volume corrected at *P* < 0.05 family-wise error (FWE). Activation outside the mPFC was thresholded at *P* < 0.05 FWE corrected for the whole brain, >10 voxels. Coordinates (*x*, *y*, and *z*) are cluster peaks from a conjunction analysis of the 4 simple effects in MNI space. *T*-values at these peaks are reported from simple contrasts of episodic conditions versus the pooled control condition, and from a conjunction analysis where the control trials were split into 4 independent baselines.

To verify the reliability of the above findings, a further analysis was conducted where trials in the Control condition were randomly split into quarters and used as 4 independent baselines for the episodic task conditions. The conjunction between these independent contrasts was calculated, which confirmed that all peaks identified in the pooled analysis were also significant when using independent Control conditions (Table 2), although the independent conjunction analysis was obviously less powerful.

### General Old versus New Effects

The next analysis investigated regions commonly activated during correct source memory judgments for old items compared with correct identification of new items, irrespective of retrieval task and study condition. This analysis was performed to assess whether there were any task-independent effects of episodic retrieval success in the mPFC, which might be expected if the mPFC mediates postretrieval processing of social information across both agentic and conceptual domains. A conjunction analysis was conducted on the individual contrasts between Agentic old > new, and Conceptual old > new, which revealed no significant activations within the mPFC ROI, but several significant clusters in the whole-brain analysis, including regions in the left inferior lateral and medial parietal cortex, left dorsolateral PFC, medial frontal cortex, bilateral anterior insula and striatum (Table 3). The reverse conjunction contrast, testing regions more activated for new than old items, revealed 2 highly significant clusters in secondary visual regions, as well as a smaller right parietal cluster (Table 3).

**Table 3:** Whole-brain fMRI activation differences between old and new items common to both episodic retrieval tasks.

Hemisphere	Region	BA	*x*	*y*	*z*	Voxels	Conjunction *T*-value
Old > new							
Left	Supramarginal gyrus/intraparietal sulcus/angular gyrus	39/40	−33	−55	40	124	7.85
Left	Middle frontal gyrus	9/46	−39	8	40	230	7.5
Left	Superior frontal gyrus/ cingulate gyrus	6/8/32	−6	17	46	125	7.41
Left	Precuneus	7	−6	−67	34	99	7.04
Left	Anterior insula	13	−27	26	1	53	6.82
Left	Basal ganglia	Striatum	−12	5	−2	16	6.04
Right	Basal ganglia	Striatum	9	5	−2	22	5.87
New > Old							
Left	Cuneus	18	−9	−94	16	128	9.14
Right	Lingual gyrus	18	12	−70	−2	78	7.47
Right	Supramarginal gyrus	40	54	−28	28	28	5.75

Notes: Presented effects are thresholded at *P* < 0.05 FWE corrected for the whole brain, >10 voxels (there were no common old/new effects in the mPFC). Coordinates (*x*, *y*, and *z*) are cluster peaks in MNI space from a conjunction analysis between 2 old/new simple contrasts within each of the episodic retrieval tasks.

### Differences Between Old Items as a Function of Self-Referential Encoding and Retrieval Task

The final, critical analysis investigated the hypothesis that mPFC would be differentially involved in self-related episodic recollection as a function of both 1) the type of self-referential processing engaged at study and 2) the retrieval test requirements. This involved contrasting old words related to the self or Obama (You > Obama) during the Conceptual recollection task, and contrasting old words spoken aloud by the participants or experimenter (Subject > Experimenter) during the Agentic recollection task. In line with previous research (Benoit et al. 2010), self-related words significantly increased ventral mPFC activation compared with other-related words in the Conceptual recollection task (one large ventral cluster with 163 voxels at *x, y, z*: −9, 53, 13, *t* = 4.67, see Fig. 2*B*; a second smaller dorsal cluster with 13 voxels at *x, y, z*: 6, 59, 31, *t* = 3.69). However, no similar self-reference effect was found on trials when participants judged whether they or the experimenter had spoken the word (there were no self > other activation differences for Agentic recollection in the mPFC). In fact, both clusters in the You > Obama contrast in the Conceptual recollection task were still significant when exclusively masked with a leniently thresholded (*P* < 0.05, uncorrected) Subject > Experimenter contrast in the Agentic recollection task, indicating that self > other mPFC activation differences were restricted to conceptual self-referential memories. A second analysis separated old items in the Agentic recollection task based on whether the participant had related the word to themselves or Obama. This analysis revealed no significant mPFC differences, showing that the self-referential effect was task dependent.

Finally, to verify that the response in the ventral mPFC was indeed qualitatively different from the dorsal mPFC activation pattern, we extracted the percent signal change values from ventral and dorsal regions and ran a region (dorsal/ventral) × old condition (Agentic Subject, Agentic Experimenter, Conceptual You, and Conceptual Obama) Analysis of variance (ANOVA) on these values. However, as the ventral peak in our main analysis was defined by a contrast that was nonorthogonal to the effect tested for in the current analysis, it would be inappropriate to extract signal from this peak because doing so might bias the significance of the results (an example of “double dipping”, see Kriegeskorte et al. 2009). Therefore, we defined the ventral peak based on a recent meta-analysis by Denny et al. (2012), which demonstrated a maximum difference between self- and other-referential processing in the ventral mPFC (coordinates: −10, 50, 6) across 48 studies. Percent signal change extracted from this ventral peak was compared with percent signal change in our dorsal peak (coordinates: −12, 59, 25, Fig. 2*A*), since the latter was defined based on an orthogonal contrast (common activation for old and new items in the episodic tasks compared with control, collapsed across Study condition) to that tested in the current ANOVA and thus not statistically biased. This analysis confirmed a significant interaction (*F*_3,51_ = 6.66, *P* = 0.001).

## Discussion

In the current study, we investigated the brain regions that support different types of self-referential processing during episodic retrieval. Previous research has revealed a functional gradient in the mPFC during nonepisodic tasks, whereby the dorsal mPFC appears to have a general role in social cognition whereas the ventral mPFC may have a specific role in conceptual self-referential processing (Northoff and Bermpohl 2004; Mitchell et al. 2006; D'Argembeau et al. 2007; Moran et al. 2011; Denny et al. 2012; Wagner et al. 2012; Martinelli et al. 2013). Based on the assumption that episodic retrieval involves a reinstatement of the neurocognitive processes that are engaged when perceiving and comprehending an initial event (Morris et al. 1977; Rugg et al. 2008), we predicted that a similar distinction would be observed during recollection. Consistent with our predictions, the dorsal mPFC was generally activated when participants attempted to retrieve social information, whereas the ventral mPFC was specifically activated during recollection of information that was previously encoded with reference to participants' conceptual self.

Dorsal mPFC activation was found for both old and new items in both episodic tasks, and for both self-encoded and other-encoded old items in comparison to the nonepisodic control condition. Importantly, these common episodic task effects on brain activity cannot be explained by similar behavioral performance for these conditions when compared with the control condition, because new and old items in the episodic tasks were associated with very different behavioral profiles. Participants were faster, more accurate and more confident when making memory judgments on new items in the episodic tasks than when making letter judgments on new items in the control task. In contrast, they were slower, less confident and less accurate for old items in the episodic tests when compared with control task performance. Thus, general episodic task-related activity in the dorsal mPFC suggests that this region can be confidently linked to episodic retrieval processes without potentially confounding behavioral differences. Furthermore, since similar activation levels were found in this region for both old and new items in the episodic tasks, this suggests that the dorsal mPFC was not engaged based on recollection success, but rather may mediate preretrieval processes that are recruited to facilitate recollection (see e.g., Rugg and Wilding 2000; Benoit et al. 2009). Activation in this region for both self- and other-encoded items is consistent with previous findings that dorsal mPFC is generally engaged during social cognition (e.g., Wagner et al. 2012; Hassabis et al. 2013).

In contrast, activation in the ventral mPFC was dependent on both the type of information initially encoded and the type of information participants were asked to retrieve. When participants were asked to retrieve whether they had related a person-descriptive word to themselves or another person, ventral mPFC was particularly engaged for items that had been encoded in relation to their concept of self compared with the concept of another person, but no similar conceptual self > other difference was found in the agentic recollection task. Nor was there an enactment-related difference in this region between items that had been spoken out loud by the participant versus items that had been spoken by the experimenter. This selective response in the ventral mPFC is highly consistent with previous findings relating this region specifically to conceptual rather than bodily self-referential processing (e.g., Powell et al. 2010), and with the more general argument that conceptual and bodily aspects of self are dissociable (e.g., Blakemore and Frith 2003; Gillihan and Farah 2005; Lind 2010; see also Williams 2010, for a somewhat different distinction).

There are several interesting aspects of this effect in the ventral mPFC. First, because it was dependent on the encoding conditions of particular stimuli, recruitment of this region appears to be contingent on successful retrieval of conceptual self-information, consistent with previous findings (e.g., Fossati et al. 2004; Benoit et al. 2010). Thus, ventral mPFC may mediate postretrieval processing of recollected information rather than preretrieval processes relating to retrieval attempts (Rugg and Wilding 2000). Second, conceptual self > other differences in the ventral mPFC were only found during the conceptual recollection task and not during the agentic recollection task, suggesting that the self-referential process mediated by this region was not automatically elicited, but rather was flexibly engaged based on task demands. Previous research on self-referential processing in nonepisodic tasks have shown that ventral mPFC activity is enhanced for conceptually self-relevant stimuli even when the task does not require explicit self-referential judgments (e.g., Rameson et al. 2010). However, such automatic effects have primarily been found for stimuli that are very strongly self-relevant, such as personal semantic facts (Moran, et al. 2009). In our task, although participants encoded person-descriptive words in relation to their conceptual self highly successfully (as judged by their subsequent accurate source memory for those words), such episodic encoding appears not to have elicited automatic self-referential processing to the same degree as did personal semantic facts in Moran et al.'s experiment.

The behavioral data showed that both self-enactment and conceptual self-referential processing resulted in enhanced recognition memory, in line with typical findings (Rogers et al. 1977; Engelkamp and Zimmer 1989). However, neither self-enactment nor conceptual self-referential processing enhanced source memory accuracy for the self-relevant source, which has sometimes been found in the previous literature (e.g., Serbun et al. 2011). The lack of source memory effects in our study is however difficult to interpret, because we were unable to correct our source memory measure for response biases (such as the “it had to be you” effect, Johnson et al. 1981), as done in previous studies (e.g., Serbun et al. 2011). Estimating response biases requires an examination of the type of errors participants make to new items, but new item accuracy was at ceiling in our data. Therefore, our behavioral source memory results are not very informative on this point since if there were self-referential effects on source accuracy in our study, these may have been obscured by response biases that we were unable to measure. Nevertheless, despite similar behavioral outcomes, the fMRI results indicate that self-referential effects due to performing an action versus relating information to one's concept of self have distinct neural underpinnings, since only the latter was associated with ventral mPFC engagement.

Interestingly, performance on the conceptual recollection task was weaker for participants who rated themselves as more similar to Obama, replicating previous research (Benoit et al. 2010). This pattern supports the view that similar self-referential processes are also applied when thinking about people considered similar to oneself (Mitchell et al. 2006; Benoit et al. 2010). That is, employing similar processes during encoding would lead to less discriminant memory traces, which, in turn, would make it more difficult to remember whether one had made the initial judgment about oneself or the similar other person.

We provided further evidence for 2 dissociable forms of self-reflection—conceptual versus agentic—by examining the fate of items that served as foils during the main memory task. Specifically, if people can intentionally orient retrieval towards either type of self-referential information, this might lead to differential incidental encoding of new information encountered during the 2 retrieval tasks. Previous research has shown that incidental encoding of new information is enhanced if that new information is tested in the same context as old information that was particularly effectively encoded during a preceding study phase. These observations have been taken to suggest that people strategically reinstate encoding processes during retrieval attempts (e.g., Jacoby, Shimizu, Daniels et al. 2005; Jacoby, Shimizu, Velanova 2005; Marsh et al. 2009; Danckert et al. 2011). In our experiment, participants were more accurate at judging whether a personality word had been related to themselves or President Obama than judging whether a word was spoken by themselves or the experimenter, suggesting that conceptual self-referential processing led to more effective encoding than agentic self-referential processing. In the final recognition test for items presented as foils in the preceding main experiment, foils were more accurately recognized if they had previously been presented with a conceptual self/other retrieval question than an agentic self/other retrieval question. This final test difference occurred despite highly similar behavioral performance for foils during their initial exposure, suggesting that it cannot be simply explained by differences in processing effort or study time during the first test.

Instead, our findings are more consistent with the view that people engaged distinct types of self-referential processing in response to the different test questions, in an attempt to strategically constrain retrieval to either conceptual or agentic self-referential information. Most previous research (e.g., Jacoby, Shimizu, Daniels et al. 2005; Jacoby, Shimizu, Velanova 2005; Marsh et al. 2009; Danckert et al. 2011; Halamish et al. 2012) has demonstrated such retrieval orientation effects on encoding using some form of level-of-processing manipulation (Craik and Tulving 1975), which typically produces very large effects on encoding. To our knowledge, ours is the first demonstration that even very subtle differences between retrieval attempts—in this case, attempting to retrieve distinct types of self-referential information—can produce differential incidental encoding of new information.

The current research has demonstrated a distinction within the mPFC during episodic recollection that is consistent with the previously proposed dorsal–ventral gradient for general social cognition versus conceptual self-referential processing (e.g., Wagner et al. 2012). However, the functional significance of this gradient remains to be determined, as the exact nature of processing or representation mediated by mPFC regions is still unclear (see e.g., Mitchell 2009). It is also not known whether the link between mPFC and social cognition is indicative of a specialized “module”, or whether the mPFC mediates more general cognitive processes that happen to be particularly engaged during these types of tasks.

According to one view, activations in the mPFC indicate that conceptual self-referential enhancements of memory are the result of a unique type of processing that is qualitatively different from general semantic processes, since the latter are typically associated with left lateral prefrontal regions (Kelley et al. 2002). Another view suggests that the concept of self is a particularly rich and elaborate semantic schema, and that relating information to this schema facilitates encoding (e.g., Kihlstrom et al. 2003). Accordingly, self-referential effects may only be *quantitatively* different from other semantic effects on encoding. Consistent with the latter view, recent evidence suggests that general schema-related memory enhancements are mediated by the mPFC even when the schema in question is neither obviously socially- nor self-relevant (reviewed in Van Kesteren et al. 2012). Thus, future research should aim to determine whether self-referential and general schema-related effects on memory involve similar recruitment of the mPFC.

An alternative line of research has focused on the role of the mPFC in value judgments during decision making. Recent research in this field has suggested that the self/other gradient in the mPFC is not fixed, but that dorsal and ventral mPFC process both self- and other-related information depending on whether that information is relevant to a currently executed or alternative, nonexecuted choice (Nicolle et al. 2012). Based on such evidence suggesting that the ventral mPFC may not support self-representations per se, D'Argembeau (2013) has suggested that this region may instead evaluate or represent the personal value or significance (i.e., the worth or importance of something for an individual) of externally and internally generated information. Since self-referential information tends to be considered more personally significant, this region tends to be more active during self-referential than other-referential processing. According to this view, the ventral mPFC activity pattern observed in the current study may be due to participants attaching particularly high personal significance to the recollection that a personality descriptive word had previously been related to their conceptual self compared with other types of recollected information.

In conclusion, our findings demonstrate a fractionation between different sub-regions within the mPFC that are engaged during recollection of different types of self-referential information, supporting the view that the self is not a unitary phenomenon. Rather, the brain regions that process information about our conceptual self appear partially nonoverlapping with the brain regions that process information about our bodily self. Whereas previous research has demonstrated this distinction during on-line processing of perceptual information in the environment, our findings show a similar dissociation when processing information retrieved from episodic long-term memory.

## Funding

This work was supported by the BBSRC [grant number BB/G014795/1] and the James S McDonnell Foundation. Funding to pay the Open Access publication charges for this article was provided by the University of Cambridge's RCUK block grant for Open Access.
